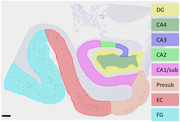# Hippocampal Amyloid‐Beta And Tau Distributions Differentially Affect Cognition In Centenarians

**DOI:** 10.1002/alz70855_096283

**Published:** 2025-12-23

**Authors:** Susan K. Rohde, Maruelle C. Luimes, Annemieke J.M. Rozemuller, Marieke J.I. Graat, Myke E. van der Hoorn, Dominique A.H. Daatselaar, Marc Hulsman, Sietske A.M Sikkes, Timothy E. Richardson, Jeroen J.M. Hoozemans, Jamie M. Walker, Henne Holstege

**Affiliations:** ^1^ Amsterdam UMC, location VUmc, Amsterdam, Netherlands, Amsterdam, Netherlands, Amsterdam, Netherlands; ^2^ Alzheimer Center Amsterdam, Department of Neurology, Amsterdam Neuroscience, Vrije Universiteit Amsterdam, Amsterdam UMC, Amsterdam, Netherlands, Amsterdam, Netherlands; ^3^ Section Genomics of Neurodegenerative Diseases and Aging, Department of Clinical Genetics, Vrije Universiteit Amsterdam, Amsterdam UMC, Amsterdam, Netherlands, Amsterdam, Netherlands; ^4^ Department of Pathology, Amsterdam Neuroscience, Amsterdam UMC, Amsterdam, Netherlands, Amsterdam, Netherlands, Amsterdam, Netherlands; ^5^ Netherlands Institute for Neuroscience, Amsterdam, Netherlands; ^6^ Department of Pathology, Amsterdam Neuroscience, Amsterdam UMC, Amsterdam, Noord‐Holland, Netherlands; ^7^ Amsterdam UMC, Amsterdam, Netherlands; ^8^ Genomics of Neurodegenerative Diseases and Aging, Human Genetics, Amsterdam UMC, Amsterdam, Netherlands; ^9^ Alzheimer Centre, Department of Neurology, Amsterdam Neuroscience, Vrije Universiteit Amsterdam, Amsterdam UMC, Amsterdam, The Netherlands, Amsterdam, Netherlands; ^10^ Alzheimer Center Amsterdam, Neurology, Vrije Universiteit Amsterdam, Amsterdam UMC, Amsterdam, Netherlands; ^11^ Icahn School of Medicine at Mount Sinai, New York, NY, USA; ^12^ Roche Pharma Research and Early Development, Basel, Switzerland; ^13^ Delft Bioinformatics Lab, Delft University of Technology, Delft, The Netherlands, Delft, Netherlands, Delft, Netherlands; ^14^ Alzheimer Center Amsterdam, Department of Neurology, Amsterdam Neuroscience, Vrije Universiteit Amsterdam, Amsterdam UMC, Amsterdam, Netherlands; ^15^ VIB‐KU Leuven Center for Brain & Disease Research, Leuven, Belgium

## Abstract

**Background:**

The hippocampus is differentially affected in Alzheimer's disease neuropathologic change (ADNC) versus primary age‐related tauopathy (PART), an amyloid‐beta (Aβ)‐independent tauopathy: the CA2/CA1 hyperphosphorylated tau (pTau)‐ratio is higher in PART, which inversely correlates with Aβ‐burden. However, as the aging brain often presents mixed rather than uniform pathologies, we questioned whether these distinct hippocampal pTau distributions persist into extreme ages and how hippocampal Aβ‐ and pTau‐distributions correlate with cognition in centenarians.

**Method:**

We quantified Aβ‐ (6F/3D) and pTau (AT8)‐burdens across eight hippocampal and parahippocampal subregions in 112 centenarians (median age 104, IQR 102‐105), alongside 11 AD (median age 84, IQR 72‐86) and 7 PART cases for comparison (median age 88, IQR 78‐92; Figure 1). We compared CA2/CA1‐pTau‐ratio in centenarians who met PART criteria (Thal phase ≤2, Braak stage I‐IV; *n* = 49) with centenarians who met ADNC criteria (intermediate/high according to NIA‐AA guidelines; Thal phase ≥3, Braak stage III‐VI; *n* = 50). Cognitive performance was assessed using 13 neuropsychological tests shortly before brain donation (median 10 months, IQR5‐14, *n* = 72). Robust linear regression models were used to associate subregional Aβ‐ and pTau‐burdens with cognitive performance, while adjusting for age, sex, and education.

**Result:**

In line with previous findings, CA2/CA1‐pTau‐ratios were higher in younger PART cases compared to AD patients (median 3.0, IQR 2.1‐3.6, min‐max 1.6‐4.2 vs. median 1.2, IQR 0.9‐1.4, min‐max 0.8‐1.4; *p* <0.001). Surprisingly, CA2/CA1‐pTau‐ratios in centenarians with PART were comparable to centenarians with ADNC (median 1.3, IQR 1.1‐2.0, min‐max 0.3‐10.8 vs. median 1.2, IQR 1.0‐1.8, min‐max 0.2‐6.2; *p* = 0.684). Accordingly, CA2/CA1‐pTau‐ratio in centenarians was unrelated to Aβ‐burden, Thal phase or Braak stage. Higher Aβ‐ and pTau‐burdens associated with lower cognition, though through different subregions: cognition associated with Aβ‐burden in the hippocampus (CA4, CA3, CA2, CA1/subiculum), whereas pTau‐burden in the parahippocampus (presubiculum, entorhinal cortex, fusiform gyrus) associated with cognition.

**Conclusion:**

In the oldest‐old, PART and ADNC are less distinguishable by determinants observed in younger individuals: centenarians with ADNC may show age‐related Aβ accumulation alongside PART‐like pTau patterns, while centenarians meeting PART criteria do not always show PART‐like pTau patterns. However, hippocampal Aβ‐burden and parahippocampal pTau‐burden associate with cognitive decline, highlighting subregional‐specific vulnerability to pathology‐driven cognitive decline.